# Tricarbon­yl(η^6^-flavone)chromium(0)

**DOI:** 10.1107/S1600536809040525

**Published:** 2009-10-10

**Authors:** Johannes H. van Tonder, Barend C. B. Bezuidenhoudt, J. Marthinus Janse van Rensburg

**Affiliations:** a Department of Chemistry, University of the Free State, PO Box 339, Bloemfontein 9300, South Africa; b Department of Pharmacology, University of Pretoria, PO Box 2034, Pretoria 0001, South Africa

## Abstract

In the title compound, [Cr(C_15_H_10_O_2_)(CO)_3_], the Cr(CO)_3_ unit exhibits a three-legged piano-stool conformation. The chromium metal centre is coordinated by the phenyl ring of the flavone ligand [Cr—(phenyl centroid) distance = 1.709 (1) Å]. The ligand is approximately planar, the dihedral angles between the γ-pyrone ring and the phenyl ring and between the γ-pyrone and the phenyl­ene ring being 2.91 (5) and 3.90 (5)°, respectively. The mol­ecular packing shows π–π stacking between the flavone ligands of neighbouring mol­ecules.

## Related literature

For the crystal structure of Cr(CO)_3_(C_15_H_12_O_2_), see: Dominique *et al.* (1999 ▶). For comparison bond distances, see: Allen (2002 ▶). For related structures, see: Zeller *et al.* (2004 ▶); Zhang *et al.* (2005 ▶); Czerwinski *et al.* (2003 ▶); Guzei & Czerwinski (2004 ▶). For the biological activity of flavonoids, see: Rice-Evans & Packer (2003 ▶).
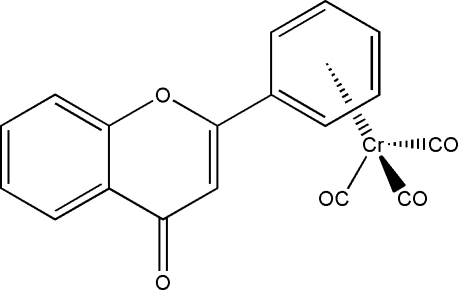


## Experimental

### 

#### Crystal data

[Cr(C_15_H_10_O_2_)(CO)_3_]
*M* *_r_* = 358.26
Triclinic, 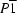

*a* = 7.2853 (2) Å
*b* = 9.6427 (3) Å
*c* = 11.6466 (4) Å
α = 78.545 (1)°
β = 79.554 (1)°
γ = 70.005 (1)°
*V* = 747.81 (4) Å^3^
*Z* = 2
Mo *K*α radiation
μ = 0.79 mm^−1^
*T* = 173 K
0.45 × 0.32 × 0.19 mm

#### Data collection

Bruker APEXII CCD diffractometer
Absorption correction: multi-scan (*SADABS*; Bruker, 2004 ▶) *T*_min_ = 0.717, *T*_max_ = 0.864
8083 measured reflections
3600 independent reflections
3062 reflections with *I* > 2σ(*I*)
*R*_int_ = 0.022

#### Refinement

*R*[*F* ^2^ > 2σ(*F* ^2^)] = 0.037
*wR*(*F* ^2^) = 0.105
*S* = 1.06
3600 reflections
205 parameters
H-atom parameters constrained
Δρ_max_ = 0.52 e Å^−3^
Δρ_min_ = −0.55 e Å^−3^

### 

Data collection: *APEX2* (Bruker, 2005 ▶); cell refinement: *SAINT-Plus* (Bruker, 2004 ▶); data reduction: *SAINT-Plus*; program(s) used to solve structure: *SIR97* (Altomare *et al.*, 1999 ▶); program(s) used to refine structure: *SHELXL97* (Sheldrick, 2008 ▶); molecular graphics: *DIAMOND* (Brandenburg & Putz, 2005 ▶); software used to prepare material for publication: *WinGX* (Farrugia, 1999 ▶).

## Supplementary Material

Crystal structure: contains datablocks global, I. DOI: 10.1107/S1600536809040525/ng2657sup1.cif

Structure factors: contains datablocks I. DOI: 10.1107/S1600536809040525/ng2657Isup2.hkl

Additional supplementary materials:  crystallographic information; 3D view; checkCIF report

## Figures and Tables

**Fig. 1.**
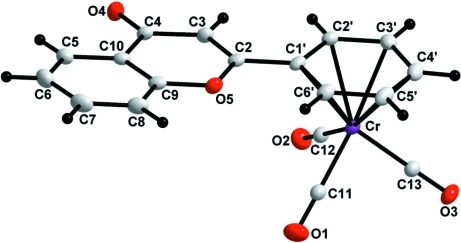
A view of (I) showing the atom-numbering scheme with displacement ellipsoids at the 30% probability level.

**Fig. 2.**
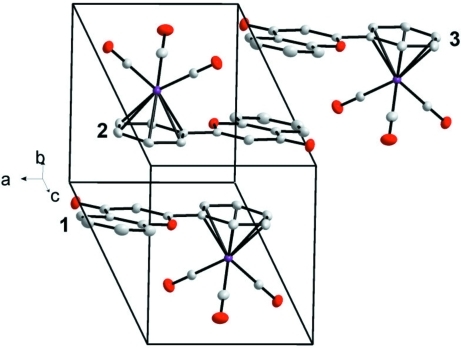
Indication of molecular packing in the unit-cell, displaying π-stacking. H-atoms are omitted for clarity. Symmetry operators 1) x; y; z. 2) 1 - x; 1 - y; 1 - z. 3) 1 + x; 1 + y; z.

## supplementary crystallographic information

### Comment

Flavanoids are an extensive group of polyphenolic compounds that occur commonly in plants. Many flavonoids are known to show biological activities such as anti-inflammatory, antibacterial and antifungal properties (Rice-Evans & Packer., 2003) The steric influence from a Cr(CO)_3_ moiety combined with the electronic alteration of an arene ring, *via* metal coordination, made the tricarbonyl(arene)chromium complexes very popular intermediates in regioselective organic synthesis (Dominique *et al.*, 1999).

In the course of our work on flavanoids we isolated and characterized the title compound, (I), [Cr(CO)_3_(C_15_H_10_O_2_)], where (C_15_H_10_O_2_) = flavone. The title compound crystallized in the triclinic space group P-1, with *Z* = 2 (Fig.1). The chromium metal centre coordinated to the phenyl ring of the flavone moiety and together with the tricarbonyl group a three-legged piano-stool conformation is exhibited. The Cr—C(arene) distances range from 2.209 (2) to 2.225 (2) Å and the chromium metal centre is displaced by 1.709 (1) Å from the B-η^6^-coordinated arene ring centre. The carbonyl groups are fairly linear with Cr—*C*(carbonyl)—O angles ranging from 179.0 (2) to 179.4 (2)°. The Cr—*C*(carbonyl) bonds of Cr—C11, Cr—C12 and Cr—C13 are 1.847 (2), 1.844 (2) and 1.842 (2) Å respectively. While the carbonyl distances of C11—O1, C12—O2 and C13—O3 are 1.153 (3), 1.155 (3) and 1.153 (3) respectively. These carbonyl distances are well within the normal range, see Allen (2002).

The phenyl ring of the flavone backbone is essentialy planar (r.m.s of fitted atoms C1'-C6' = 0.0083 Å). The γ-pyrone and the benzene ring of the flavone skeleton is in the same plane as the phenyl ring. A small molecular disorder is displayed by the dihedral angle of 2.91° between the γ-pyrone and the phenyl ring and the torsion angle of -178.78 (15)° formed by atoms C2'-C1'-C2—O5. The benzene ring is lifted out of the molecular plane, with a 3.90 (5)° dihedral angle between the γ-pyrone and the benzene ring. Other molecular geometrical parameters is in good agreement with literature values, see Allen (2002). Selected geometrical parameters is presented in Table 1.

The molecular packing displays two types of ligand to ligand π-π stacking. This is on opposite sides of the 2-phenylchromane backbone (Fig.2). One type of packing is where the tricarbonyl-metal moieties of neighbouring molecules are directed away from one another resulting in a ligand to ligand π-π stacking between the γ-pyrone and phenyl rings, with a plane to plane distance of 3.354 Å. The other type of π-π stacking is between the γ-pyrone and benzene rings of neighbouring molecules, with a plane to plane distance of 3.418 Å, this π-π stacking is secondarily stabilized by soft contacts between O1···H5 [2.761 (3) Å] and a O1···H5—C5 angle of 129.8 (1)°.

### Experimental

A solution of flavone (1.01 g; 4.5 mmol) and Cr(CO)_6_ (1.0 g; 4.6 mmol; 1 eq.) in Bu_2_O:THF (9:1; 10 ml per 100 mg Cr(CO)_6_) was degassed with argon, using standard Schlenk techniques, and refluxed (48 h) under an oxygen free atmosphere. The reaction mixture was cooled to room temperature and the solvent evaporated *in vacuo*. Purification through flash column-chromatography yielded tricarbonyl(B-η^6^-flavone)chromium(0) (0.70 g; 42.9%) as an orange solid. Recrystallization from diethyl ether yielded orange crystals suitable for single-crystal diffraction data collection.

*R_f_* 0.11 (H:A:DCM; 7:1:2); Mp 160.6 °C; Note: A, B and C-ring labelling refers to the benzene, phenyl and γ-pyrone rings respectively. ^1^H NMR (600 MHz, CDCl_3_) δ p.p.m. 8.21 (1*H*, d, J = 7.91 Hz, H-5), 7.72 (1*H*, dd, J = 7.53, 7.91 Hz, H-6), 7.54 (1*H*, d, J = 8.28 Hz, H-8), 7.44 (1*H*, dd, J = 7.53, 8.28 Hz, H-8), 6.61 (1*H*, s, H-3), 6.00 (2*H*, d, J = 6.38 Hz, H-2' and H-6'), 5.57 (1*H*, t, J = 6.14 Hz, H-4'), 5.41 (2*H*, dd, J = 6.14, 6.38 Hz, H-3' and H-5'); ^13^C NMR (151 MHz, CDCl_3_) δ p.p.m. 89.91 (C-3' and C-5'), 91.04 (C-2' and C-6'), 93.77 (C-4'), 94.98, 107.38 (C-3), 118.08 (C-8), 124.0, 125.79 (C-5 or C-7), 125.92 (C-5 or C-7), 134.33 (C-6), 156.13, 161.02, 177.56, 231.08(–Cr(CO)3); MS (MS Scheme 4) m/*z* 358 (*M*+, 4.4), 330 (0.9), 302 (2.1), 274 (11.3), 239 (2.2), 223 (100.0), 210 (0.6), 183 (2.6), 155 (3.5), 121 (29.0), 103 (4.7).

### Refinement

The H atoms were positioned geometrically and refined using a riding model with fixed C—H distances of 0.93 Å (CH) [*U*_iso_(H) = 1.2*U*_eq_] and 0.96 Å.

The highest density peak is 0.36 located 0.76 Å from C3 and the deepest hole is -0.35 located at 0.61 Å from Cr.

### Crystal data

**Table:**

[Cr(C_15_H_10_O_2_)(CO)_3_]	*Z* = 2
*M**_r_* = 358.26	*F*(000) = 364
Triclinic, *P*1	*D*_x_ = 1.591 Mg m^−^^3^
Hall symbol: -P 1	Mo *K*α radiation, λ = 0.71073 Å
*a* = 7.2853 (2) Å	Cell parameters from 3694 reflections
*b* = 9.6427 (3) Å	θ = 2.3–28.2°
*c* = 11.6466 (4) Å	µ = 0.79 mm^−^^1^
α = 78.545 (1)°	*T* = 173 K
β = 79.554 (1)°	Irregular, orange
γ = 70.005 (1)°	0.45 × 0.32 × 0.19 mm
*V* = 747.81 (4) Å^3^	

### Data collection

**Table:**

Bruker APEXII CCD diffractometer	3600 independent reflections
Radiation source: fine-focus sealed tube	3062 reflections with *I* > 2σ(*I*)
graphite	*R*_int_ = 0.022
φ and ω scans	θ_max_ = 28°, θ_min_ = 1.8°
Absorption correction: multi-scan (*SADABS*; Bruker, 2004)	*h* = −9→8
*T*_min_ = 0.717, *T*_max_ = 0.864	*k* = −12→12
8083 measured reflections	*l* = −15→14

### Refinement

**Table:**

Refinement on *F*^2^	0 restraints
Least-squares matrix: full	H-atom parameters constrained
*R*[*F*^2^ > 2σ(*F*^2^)] = 0.037	*w* = 1/[σ^2^(*F*_o_^2^) + (0.0513*P*)^2^ + 0.3895*P*] where *P* = (*F*_o_^2^ + 2*F*_c_^2^)/3
*wR*(*F*^2^) = 0.105	(Δ/σ)_max_ = 0.001
*S* = 1.06	Δρ_max_ = 0.52 e Å^−^^3^
3600 reflections	Δρ_min_ = −0.55 e Å^−^^3^
205 parameters	

### Special details

**Table:**

Experimental. The intensity data was collected on a Bruker Apex II CCD diffractometer using an exposure time of 10 s/frame. The 509 frames were collected with a frame width of 0.5° covering up to θ = 28° with 99.8% completeness accomplished.
Geometry. All e.s.d.'s (except the e.s.d. in the dihedral angle between two l.s. planes) are estimated using the full covariance matrix. The cell e.s.d.'s are taken into account individually in the estimation of e.s.d.'s in distances, angles and torsion angles; correlations between e.s.d.'s in cell parameters are only used when they are defined by crystal symmetry. An approximate (isotropic) treatment of cell e.s.d.'s is used for estimating e.s.d.'s involving l.s. planes.

### Fractional atomic coordinates and isotropic or equivalent isotropic displacement parameters (Å^2^)

**Table:**

	*x*	*y*	*z*	*U*_iso_*/*U*_eq_	
Cr	0.44722 (4)	0.35008 (3)	0.85182 (3)	0.02434 (11)	
O5	0.6342 (2)	0.24078 (16)	0.52790 (12)	0.0282 (3)	
O4	1.1793 (2)	0.29078 (17)	0.44675 (15)	0.0349 (4)	
O3	0.2698 (2)	0.3373 (2)	1.10514 (14)	0.0421 (4)	
C2	0.6832 (3)	0.3367 (2)	0.57827 (16)	0.0241 (4)	
C3	0.8617 (3)	0.3562 (2)	0.55295 (18)	0.0267 (4)	
H3	0.8879	0.4247	0.591	0.032*	
C10	0.9548 (3)	0.1789 (2)	0.41199 (17)	0.0249 (4)	
C13	0.3382 (3)	0.3412 (2)	1.00763 (19)	0.0292 (3)	
O1	0.4968 (3)	0.02553 (19)	0.86574 (17)	0.0499 (5)	
C9	0.7671 (3)	0.1667 (2)	0.44291 (18)	0.0273 (4)	
C12	0.6912 (3)	0.2990 (2)	0.90154 (18)	0.0292 (3)	
O2	0.8445 (2)	0.26801 (19)	0.93196 (16)	0.0425 (3)	
C5	1.0793 (3)	0.1028 (2)	0.32237 (19)	0.0311 (4)	
H5	1.2083	0.1097	0.2999	0.037*	
C2'	0.5336 (3)	0.5228 (2)	0.72042 (18)	0.0278 (4)	
H2'	0.653	0.5461	0.7082	0.033*	
C1'	0.5171 (3)	0.4148 (2)	0.65939 (17)	0.0257 (4)	
C4	1.0145 (3)	0.2762 (2)	0.46978 (17)	0.0255 (4)	
C6'	0.3370 (3)	0.3817 (2)	0.68058 (18)	0.0306 (4)	
H6'	0.3226	0.3099	0.6406	0.037*	
C11	0.4775 (3)	0.1501 (2)	0.86148 (18)	0.02916 (17)	
C3'	0.3758 (3)	0.5956 (2)	0.79855 (19)	0.0317 (5)	
H3'	0.3872	0.67	0.8369	0.038*	
C8	0.7000 (4)	0.0807 (2)	0.3874 (2)	0.0348 (5)	
H8	0.5712	0.0733	0.4094	0.042*	
C4'	0.2009 (3)	0.5587 (3)	0.8202 (2)	0.0354 (5)	
H4'	0.0964	0.6049	0.876	0.042*	
C5'	0.1799 (3)	0.4541 (3)	0.7601 (2)	0.0361 (5)	
H5'	0.0597	0.4319	0.7728	0.043*	
C7	0.8262 (4)	0.0070 (3)	0.3002 (2)	0.0398 (5)	
H7	0.7839	−0.0528	0.2617	0.048*	
C6	1.0148 (4)	0.0180 (2)	0.2667 (2)	0.0381 (5)	
H6	1.099	−0.0332	0.2055	0.046*	

### Atomic displacement parameters (Å^2^)

**Table:**

	*U*^11^	*U*^22^	*U*^33^	*U*^12^	*U*^13^	*U*^23^
Cr	0.01993 (17)	0.02901 (19)	0.02080 (17)	−0.00703 (13)	−0.00249 (12)	0.00247 (12)
O5	0.0261 (7)	0.0341 (7)	0.0279 (7)	−0.0153 (6)	−0.0025 (6)	−0.0027 (6)
O4	0.0274 (8)	0.0387 (8)	0.0435 (9)	−0.0175 (7)	0.0046 (7)	−0.0124 (7)
O3	0.0351 (9)	0.0547 (10)	0.0292 (8)	−0.0129 (8)	0.0047 (7)	−0.0001 (7)
C2	0.0270 (10)	0.0263 (9)	0.0193 (9)	−0.0107 (8)	−0.0069 (8)	0.0040 (7)
C3	0.0283 (10)	0.0280 (10)	0.0264 (10)	−0.0130 (8)	−0.0034 (8)	−0.0029 (8)
C10	0.0284 (10)	0.0215 (9)	0.0242 (9)	−0.0093 (8)	−0.0069 (8)	0.0033 (7)
C13	0.0240 (7)	0.0315 (7)	0.0279 (7)	−0.0069 (6)	−0.0014 (6)	0.0000 (6)
O1	0.0698 (13)	0.0385 (9)	0.0470 (10)	−0.0233 (9)	−0.0162 (10)	0.0000 (8)
C9	0.0333 (11)	0.0253 (9)	0.0247 (9)	−0.0127 (8)	−0.0075 (8)	0.0030 (8)
C12	0.0240 (7)	0.0315 (7)	0.0279 (7)	−0.0069 (6)	−0.0014 (6)	0.0000 (6)
O2	0.0299 (6)	0.0450 (6)	0.0502 (6)	−0.0059 (7)	−0.0131 (7)	−0.0045 (8)
C5	0.0355 (11)	0.0258 (10)	0.0305 (10)	−0.0085 (9)	−0.0039 (9)	−0.0034 (8)
C2'	0.0260 (10)	0.0272 (10)	0.0264 (10)	−0.0083 (8)	−0.0029 (8)	0.0044 (8)
C1'	0.0222 (9)	0.0312 (10)	0.0199 (9)	−0.0083 (8)	−0.0037 (7)	0.0052 (7)
C4	0.0284 (10)	0.0241 (9)	0.0244 (9)	−0.0116 (8)	−0.0020 (8)	0.0001 (7)
C6'	0.0258 (10)	0.0405 (12)	0.0239 (10)	−0.0111 (9)	−0.0070 (8)	0.0036 (8)
C11	0.0240 (2)	0.03146 (19)	0.02793 (16)	−0.0069 (6)	−0.0014 (6)	0.0000 (6)
C3'	0.0316 (11)	0.0271 (10)	0.0284 (10)	−0.0026 (8)	−0.0039 (9)	0.0022 (8)
C8	0.0392 (12)	0.0337 (11)	0.0379 (12)	−0.0180 (10)	−0.0123 (10)	−0.0014 (9)
C4'	0.0249 (11)	0.0385 (12)	0.0302 (11)	0.0005 (9)	−0.0026 (9)	0.0039 (9)
C5'	0.0190 (10)	0.0506 (13)	0.0312 (11)	−0.0079 (9)	−0.0061 (8)	0.0081 (10)
C7	0.0534 (15)	0.0326 (11)	0.0410 (13)	−0.0176 (11)	−0.0167 (11)	−0.0055 (10)
C6	0.0457 (14)	0.0303 (11)	0.0368 (12)	−0.0072 (10)	−0.0060 (10)	−0.0095 (9)

### Geometric parameters (Å, °)

**Table:**

Cr—C13	1.842 (2)	C9—C8	1.396 (3)
Cr—C12	1.844 (2)	C12—O2	1.155 (3)
Cr—C11	1.847 (2)	C5—C6	1.376 (3)
Cr—C2'	2.206 (2)	C5—H5	0.95
Cr—C4'	2.209 (2)	C2'—C3'	1.402 (3)
Cr—C6'	2.211 (2)	C2'—C1'	1.420 (3)
Cr—C1'	2.2180 (19)	C2'—H2'	0.95
Cr—C5'	2.221 (2)	C1'—C6'	1.422 (3)
Cr—C3'	2.225 (2)	C6'—C5'	1.407 (3)
O5—C2	1.358 (2)	C6'—H6'	0.95
O5—C9	1.374 (3)	C3'—C4'	1.404 (3)
O4—C4	1.232 (2)	C3'—H3'	0.95
O3—C13	1.153 (3)	C8—C7	1.373 (3)
C2—C3	1.349 (3)	C8—H8	0.95
C2—C1'	1.473 (3)	C4'—C5'	1.399 (3)
C3—C4	1.446 (3)	C4'—H4'	0.95
C3—H3	0.95	C5'—H5'	0.95
C10—C9	1.388 (3)	C7—C6	1.393 (4)
C10—C5	1.399 (3)	C7—H7	0.95
C10—C4	1.469 (3)	C6—H6	0.95
O1—C11	1.153 (3)		
			
C13—Cr—C12	88.71 (9)	C6—C5—H5	120
C13—Cr—C11	88.80 (9)	C10—C5—H5	120
C12—Cr—C11	89.60 (9)	C3'—C2'—C1'	120.78 (19)
C13—Cr—C2'	134.26 (9)	C3'—C2'—Cr	72.30 (12)
C12—Cr—C2'	86.71 (8)	C1'—C2'—Cr	71.73 (11)
C11—Cr—C2'	136.58 (9)	C3'—C2'—H2'	119.6
C13—Cr—C4'	85.97 (9)	C1'—C2'—H2'	119.6
C12—Cr—C4'	135.57 (9)	Cr—C2'—H2'	128.7
C11—Cr—C4'	134.24 (9)	C2'—C1'—C6'	118.15 (19)
C2'—Cr—C4'	66.83 (8)	C2'—C1'—C2	121.01 (18)
C13—Cr—C6'	135.41 (9)	C6'—C1'—C2	120.83 (18)
C12—Cr—C6'	135.54 (9)	C2'—C1'—Cr	70.84 (11)
C11—Cr—C6'	86.67 (9)	C6'—C1'—Cr	70.99 (11)
C2'—Cr—C6'	67.00 (8)	C2—C1'—Cr	128.43 (13)
C4'—Cr—C6'	66.69 (9)	O4—C4—C3	123.10 (18)
C13—Cr—C1'	165.52 (8)	O4—C4—C10	122.58 (19)
C12—Cr—C1'	101.05 (8)	C3—C4—C10	114.29 (17)
C11—Cr—C1'	101.82 (8)	C5'—C6'—C1'	120.8 (2)
C2'—Cr—C1'	37.43 (8)	C5'—C6'—Cr	71.90 (12)
C4'—Cr—C1'	79.57 (8)	C1'—C6'—Cr	71.55 (11)
C6'—Cr—C1'	37.46 (7)	C5'—C6'—H6'	119.6
C13—Cr—C5'	101.29 (9)	C1'—C6'—H6'	119.6
C12—Cr—C5'	165.57 (9)	Cr—C6'—H6'	129.4
C11—Cr—C5'	100.88 (9)	O1—C11—Cr	179.00 (19)
C2'—Cr—C5'	78.86 (8)	C2'—C3'—C4'	120.1 (2)
C4'—Cr—C5'	36.82 (9)	C2'—C3'—Cr	70.83 (12)
C6'—Cr—C5'	37.02 (8)	C4'—C3'—Cr	70.90 (12)
C1'—Cr—C5'	67.29 (8)	C2'—C3'—H3'	119.9
C13—Cr—C3'	100.70 (9)	C4'—C3'—H3'	119.9
C12—Cr—C3'	101.88 (9)	Cr—C3'—H3'	131
C11—Cr—C3'	165.16 (9)	C7—C8—C9	117.8 (2)
C2'—Cr—C3'	36.87 (8)	C7—C8—H8	121.1
C4'—Cr—C3'	36.92 (8)	C9—C8—H8	121.1
C6'—Cr—C3'	78.55 (8)	C5'—C4'—C3'	120.2 (2)
C1'—Cr—C3'	67.01 (8)	C5'—C4'—Cr	72.06 (13)
C5'—Cr—C3'	66.27 (9)	C3'—C4'—Cr	72.17 (12)
C2—O5—C9	118.91 (15)	C5'—C4'—H4'	119.9
C3—C2—O5	122.59 (18)	C3'—C4'—H4'	119.9
C3—C2—C1'	126.27 (18)	Cr—C4'—H4'	128
O5—C2—C1'	111.14 (16)	C4'—C5'—C6'	119.9 (2)
C2—C3—C4	122.13 (18)	C4'—C5'—Cr	71.11 (12)
C2—C3—H3	118.9	C6'—C5'—Cr	71.08 (11)
C4—C3—H3	118.9	C4'—C5'—H5'	120
C9—C10—C5	118.77 (19)	C6'—C5'—H5'	120
C9—C10—C4	119.53 (19)	Cr—C5'—H5'	130.3
C5—C10—C4	121.63 (18)	C8—C7—C6	121.5 (2)
O3—C13—Cr	179.21 (19)	C8—C7—H7	119.2
O5—C9—C10	122.38 (17)	C6—C7—H7	119.2
O5—C9—C8	115.70 (19)	C5—C6—C7	120.0 (2)
C10—C9—C8	121.9 (2)	C5—C6—H6	120
O2—C12—Cr	179.4 (2)	C7—C6—H6	120
C6—C5—C10	119.9 (2)		
			
C9—O5—C2—C3	−3.8 (3)	C11—Cr—C6'—C5'	113.02 (14)
C9—O5—C2—C1'	175.14 (15)	C2'—Cr—C6'—C5'	−102.38 (15)
O5—C2—C3—C4	0.1 (3)	C4'—Cr—C6'—C5'	−28.79 (13)
C1'—C2—C3—C4	−178.63 (17)	C1'—Cr—C6'—C5'	−132.57 (19)
C2—O5—C9—C10	4.3 (3)	C3'—Cr—C6'—C5'	−65.58 (14)
C2—O5—C9—C8	−174.35 (17)	C13—Cr—C6'—C1'	160.68 (13)
C5—C10—C9—O5	−178.36 (17)	C12—Cr—C6'—C1'	−28.37 (18)
C4—C10—C9—O5	−1.2 (3)	C11—Cr—C6'—C1'	−114.41 (13)
C5—C10—C9—C8	0.2 (3)	C2'—Cr—C6'—C1'	30.19 (12)
C4—C10—C9—C8	177.37 (18)	C4'—Cr—C6'—C1'	103.78 (14)
C9—C10—C5—C6	0.0 (3)	C5'—Cr—C6'—C1'	132.57 (19)
C4—C10—C5—C6	−177.10 (19)	C3'—Cr—C6'—C1'	66.99 (13)
C13—Cr—C2'—C3'	−29.87 (18)	C1'—C2'—C3'—C4'	2.1 (3)
C12—Cr—C2'—C3'	−114.84 (14)	Cr—C2'—C3'—C4'	−52.98 (17)
C11—Cr—C2'—C3'	159.22 (14)	C1'—C2'—C3'—Cr	55.03 (16)
C4'—Cr—C2'—C3'	28.54 (13)	C13—Cr—C3'—C2'	158.72 (13)
C6'—Cr—C2'—C3'	101.93 (14)	C12—Cr—C3'—C2'	67.79 (14)
C1'—Cr—C2'—C3'	132.14 (18)	C11—Cr—C3'—C2'	−72.2 (4)
C5'—Cr—C2'—C3'	65.10 (13)	C4'—Cr—C3'—C2'	−133.0 (2)
C13—Cr—C2'—C1'	−162.01 (13)	C6'—Cr—C3'—C2'	−66.76 (13)
C12—Cr—C2'—C1'	113.01 (13)	C1'—Cr—C3'—C2'	−29.31 (12)
C11—Cr—C2'—C1'	27.07 (17)	C5'—Cr—C3'—C2'	−103.55 (14)
C4'—Cr—C2'—C1'	−103.61 (13)	C13—Cr—C3'—C4'	−68.25 (15)
C6'—Cr—C2'—C1'	−30.22 (12)	C12—Cr—C3'—C4'	−159.19 (14)
C5'—Cr—C2'—C1'	−67.04 (12)	C11—Cr—C3'—C4'	60.8 (4)
C3'—Cr—C2'—C1'	−132.14 (18)	C2'—Cr—C3'—C4'	133.0 (2)
C3'—C2'—C1'—C6'	−0.5 (3)	C6'—Cr—C3'—C4'	66.26 (13)
Cr—C2'—C1'—C6'	54.75 (15)	C1'—Cr—C3'—C4'	103.72 (14)
C3'—C2'—C1'—C2	−179.27 (17)	C5'—Cr—C3'—C4'	29.48 (13)
Cr—C2'—C1'—C2	−123.97 (17)	O5—C9—C8—C7	178.69 (19)
C3'—C2'—C1'—Cr	−55.30 (17)	C10—C9—C8—C7	0.0 (3)
C3—C2—C1'—C2'	0.1 (3)	C2'—C3'—C4'—C5'	−2.9 (3)
O5—C2—C1'—C2'	−178.79 (16)	Cr—C3'—C4'—C5'	−55.84 (18)
C3—C2—C1'—C6'	−178.62 (18)	C2'—C3'—C4'—Cr	52.95 (17)
O5—C2—C1'—C6'	2.5 (2)	C13—Cr—C4'—C5'	−114.93 (14)
C3—C2—C1'—Cr	−89.2 (2)	C12—Cr—C4'—C5'	161.05 (14)
O5—C2—C1'—Cr	91.89 (19)	C11—Cr—C4'—C5'	−30.55 (19)
C13—Cr—C1'—C2'	62.2 (4)	C2'—Cr—C4'—C5'	102.77 (14)
C12—Cr—C1'—C2'	−69.43 (13)	C6'—Cr—C4'—C5'	28.94 (13)
C11—Cr—C1'—C2'	−161.36 (12)	C1'—Cr—C4'—C5'	65.85 (13)
C4'—Cr—C1'—C2'	65.30 (12)	C3'—Cr—C4'—C5'	131.3 (2)
C6'—Cr—C1'—C2'	130.39 (18)	C13—Cr—C4'—C3'	113.80 (14)
C5'—Cr—C1'—C2'	101.66 (14)	C12—Cr—C4'—C3'	29.78 (19)
C3'—Cr—C1'—C2'	28.90 (12)	C11—Cr—C4'—C3'	−161.82 (14)
C13—Cr—C1'—C6'	−68.2 (4)	C2'—Cr—C4'—C3'	−28.50 (13)
C12—Cr—C1'—C6'	160.18 (13)	C6'—Cr—C4'—C3'	−102.33 (14)
C11—Cr—C1'—C6'	68.24 (14)	C1'—Cr—C4'—C3'	−65.42 (13)
C2'—Cr—C1'—C6'	−130.39 (18)	C5'—Cr—C4'—C3'	−131.3 (2)
C4'—Cr—C1'—C6'	−65.09 (13)	C3'—C4'—C5'—C6'	2.2 (3)
C5'—Cr—C1'—C6'	−28.73 (13)	Cr—C4'—C5'—C6'	−53.67 (18)
C3'—Cr—C1'—C6'	−101.49 (14)	C3'—C4'—C5'—Cr	55.89 (17)
C13—Cr—C1'—C2	177.0 (3)	C1'—C6'—C5'—C4'	−0.7 (3)
C12—Cr—C1'—C2	45.44 (19)	Cr—C6'—C5'—C4'	53.69 (18)
C11—Cr—C1'—C2	−46.49 (19)	C1'—C6'—C5'—Cr	−54.40 (17)
C2'—Cr—C1'—C2	114.9 (2)	C13—Cr—C5'—C4'	67.28 (14)
C4'—Cr—C1'—C2	−179.83 (19)	C12—Cr—C5'—C4'	−65.8 (4)
C6'—Cr—C1'—C2	−114.7 (2)	C11—Cr—C5'—C4'	158.24 (14)
C5'—Cr—C1'—C2	−143.5 (2)	C2'—Cr—C5'—C4'	−66.03 (13)
C3'—Cr—C1'—C2	143.77 (19)	C6'—Cr—C5'—C4'	−132.4 (2)
C2—C3—C4—O4	−178.92 (19)	C1'—Cr—C5'—C4'	−103.38 (14)
C2—C3—C4—C10	2.9 (3)	C3'—Cr—C5'—C4'	−29.55 (13)
C9—C10—C4—O4	179.48 (18)	C13—Cr—C5'—C6'	−160.28 (13)
C5—C10—C4—O4	−3.4 (3)	C12—Cr—C5'—C6'	66.6 (4)
C9—C10—C4—C3	−2.3 (3)	C11—Cr—C5'—C6'	−69.33 (14)
C5—C10—C4—C3	174.81 (18)	C2'—Cr—C5'—C6'	66.40 (13)
C2'—C1'—C6'—C5'	−0.1 (3)	C4'—Cr—C5'—C6'	132.4 (2)
C2—C1'—C6'—C5'	178.61 (18)	C1'—Cr—C5'—C6'	29.05 (12)
Cr—C1'—C6'—C5'	54.56 (17)	C3'—Cr—C5'—C6'	102.88 (14)
C2'—C1'—C6'—Cr	−54.68 (16)	C9—C8—C7—C6	−0.5 (3)
C2—C1'—C6'—Cr	124.05 (17)	C10—C5—C6—C7	−0.4 (3)
C13—Cr—C6'—C5'	28.11 (19)	C8—C7—C6—C5	0.7 (4)
C12—Cr—C6'—C5'	−160.94 (14)		
